# Distribution of multiunit pitch responses recorded intracranially from human auditory cortex

**DOI:** 10.1093/cercor/bhad186

**Published:** 2023-05-26

**Authors:** Joel I Berger, Phillip E Gander, Yukiko Kikuchi, Christopher I Petkov, Sukhbinder Kumar, Christopher Kovach, Hiroyuki Oya, Hiroto Kawasaki, Matthew A Howard, Timothy D Griffiths

**Affiliations:** Department of Neurosurgery, 1800 JPP, 200 Hawkins Drive, University of Iowa, Iowa City, IA 52242, United States; Department of Neurosurgery, 1800 JPP, 200 Hawkins Drive, University of Iowa, Iowa City, IA 52242, United States; Biosciences Institute, Newcastle upon Tyne NE2 4HH, United Kingdom; Department of Neurosurgery, 1800 JPP, 200 Hawkins Drive, University of Iowa, Iowa City, IA 52242, United States; Biosciences Institute, Newcastle upon Tyne NE2 4HH, United Kingdom; Department of Neurosurgery, 1800 JPP, 200 Hawkins Drive, University of Iowa, Iowa City, IA 52242, United States; Department of Neurosurgery, 1800 JPP, 200 Hawkins Drive, University of Iowa, Iowa City, IA 52242, United States; Department of Neurosurgery, 1800 JPP, 200 Hawkins Drive, University of Iowa, Iowa City, IA 52242, United States; Department of Neurosurgery, 1800 JPP, 200 Hawkins Drive, University of Iowa, Iowa City, IA 52242, United States; Department of Neurosurgery, 1800 JPP, 200 Hawkins Drive, University of Iowa, Iowa City, IA 52242, United States; Biosciences Institute, Newcastle upon Tyne NE2 4HH, United Kingdom

**Keywords:** intracranial, iEEG, sensory, perception, harmonic

## Abstract

The perception of pitch is a fundamental percept, which is mediated by the auditory system, requiring the abstraction of stimulus properties related to the spectro-temporal structure of sound. Despite its importance, there is still debate as to the precise areas responsible for its encoding, which may be due to species differences or differences in the recording measures and choices of stimuli used in previous studies. Moreover, it was unknown whether the human brain contains pitch neurons and how distributed such neurons might be. Here, we present the first study to measure multiunit neural activity in response to pitch stimuli in the auditory cortex of intracranially implanted humans. The stimulus sets were regular-interval noise with a pitch strength that is related to the temporal regularity and a pitch value determined by the repetition rate and harmonic complexes. Specifically, we demonstrate reliable responses to these different pitch-inducing paradigms that are distributed throughout Heschl’s gyrus, rather than being localized to a particular region, and this finding was evident regardless of the stimulus presented. These data provide a bridge across animal and human studies and aid our understanding of the processing of a critical percept associated with acoustic stimuli.

## Introduction

Pitch is a fundamental percept that is a critical aspect of music, voice perception, and sound segregation. Pitch is a perceptual attribute that can be associated with a variety of acoustic stimuli, and there has been a prolonged debate about the specific relationship between the time-domain and frequency-domain structures of acoustic stimuli and the pitch percept (Oxenham 2022). Pitch can be thought of as a perceptual attribute that has to be abstracted from sensory stimuli. A neural mechanism that represents the pitch percept should represent that percept irrespective of the sensory stimulus from which the pitch is abstracted and should only occur in regions of stimulus feature space associated with pitch: In terms of the repetition rate of stimuli, the value should be above the lower limit of pitch (about 30 Hz in humans; Krumbholz et al. 2000). Previous studies of the neural basis for pitch perception employed different categories of pitch-inducing stimuli. These include regular interval noise (RIN): noise that is iteratively delayed by a fixed time interval and added to the undelayed noise, resulting in a temporal regularity which increases the perceived salience of pitch as the number of iterations increases (Yost 1996a, 1996b; Wiegrebe et al. 1998). This stimulus allows the control of the long-term spectrum of the sound. Other studies have used harmonic complexes. Harmonics from 1 to 10 are resolved by the cochlea and are associated with strong pitch, while harmonics >10 contribute to a less salient pitch (Houtsma and Smurzynski 1990; Carlyon and Shackleton 1994). A variety of other pitch stimuli have also been used, including Huggins pitch produced by creating a phase difference between the ears for a particular noise band (e.g. Puschmann et al. 2010).

Human functional imaging studies based on fMRI to measure BOLD responses as an index of ensemble activity have compared pitch-associated stimuli with control stimuli (Griffiths et al. 2001; Patterson et al. 2002; Hall et al. 2006; Hall and Plack 2007; Puschmann et al. 2010). These studies have suggested a region in the nonprimary cortex in lateral HG and adjacent areas, which shows a greater activation to pitch, although other neuroimaging studies show more distributed pitch representations in the auditory cortex (Hall and Plack 2009; Barker et al. 2012).

The neurophysiological basis for pitch has been addressed in nonhuman animals (Bendor and Wang 2005). Employing strict criteria for the determination of pitch-sensitive single neurons in marmosets, Bendor and Wang demonstrated the selectivity of responses in a region overlapping anterolateral A1 and belt regions of auditory cortex, which is consistent with the idea of pitch only being processed in a selective area of auditory cortex (see also Feng and Wang 2017). However, recent nonhuman primate and human studies in which pitch responsiveness was defined based on responses to different pitch-associated stimuli with values above the lower limit of pitch have shown more widespread pitch-associated responses. These studies have been based on single-unit activity and multiunit activity (MUA) and local field potential (LFP) responses in macaques (Kikuchi et al. 2019) and LFPs in humans (Griffiths et al. 2010; Gander et al. 2019).

In this study, we address the neural basis at the level of MUA recorded from human auditory cortex using 3 paradigms employing pitch-associated stimuli: The first paradigm examined responses to RIN stimuli in which the number of iterations and associated salience was varied (“RIN iterations”); the second paradigm examined responses to different delays in the RIN stimulus and associated repetition rate and pitch value (“RIN delays”); and the third paradigm involved harmonic complex tones (“Harmonic Complex”). This is, to our knowledge, the first human study to examine neuronal spiking activity directly in response to pitch-associated stimuli, as opposed to LFPs or BOLD activity. It is important to examine this level, as most human studies at present utilize fMRI, with only a few examining LFPs in human intracranial recording studies, and BOLD activity may not correlate well with MUA (Logothetis et al. 2001). Moreover, MUA allows for a more direct comparison with animal neuronal studies of pitch, all of which use spiking activity in neurons as a primary response measure. The recordings from human primary and nonprimary areas allow us to test the hypothesis that responses associated with the pitch percept are localized in 1 cortical area. We also wished to examine whether different pitch-associated stimuli resulted in different spatial specificity as a possible basis for the lack of concordance between the various studies. Our intention was to assess whether the pitch associated with 1 paradigm resulted in a selective region of responsiveness.

## Materials and methods

### Subjects

Seven neurosurgical epilepsy patients (6 males, 1 females, all right-handed) were implanted with electrodes for the clinical purposes of identifying candidate regions of seizure foci. Sessions that were recorded within an hour of an epileptic seizure were excluded from analysis to avoid any potential confounds. Sites that were confirmed by epileptologists to be in the seizure region, or from tissue that was later resected, were excluded from analysis. Pure tone average hearing (0.5, 1, 2, and 4 kHz) was in the normal clinical range (<25 dB HL). High impedance contacts were used for research purposes, and patients consented for the procedure as part of the clinical care with implanted hybrid clinical-research electrodes, with the option to rescind their consent at any time. Research protocols were examined and were approved by the University of Iowa Institutional Review Board. Data were examined retrospectively, as these data were collected between 2007 and 2011, so knowledge of the presence of unit activity on the electrodes was not known at the time of recording and paradigms could not be adjusted based on this.

### Electrodes and recording setup

Subjects remained in an electromagnetically shielded facility for the duration of the recordings. Electrodes included in analyses here were a hybrid clinical-research type (Howard et al. 1996), with 14 exposed high-impedance contacts (70–300 kΩ) along the shaft, implanted along the long axis of Heschl’s gyrus (HG). All referencing was performed online, with ground and reference contacts located on the same electrode shaft. Data were recorded using a TDT-RZ2 system (Tucker-Davis Technologies), with a sampling rate of either 12,207 or 24,414 Hz. Precise electrode locations were confirmed by determining the Montreal Neurological Institute (MNI) coordinates from electrode tracts on each individual subject’s MRI (see Gander et al. 2019 for further details of this process).

### Experimental design and statistical analyses

#### Auditory stimuli

**Fig. 1:**
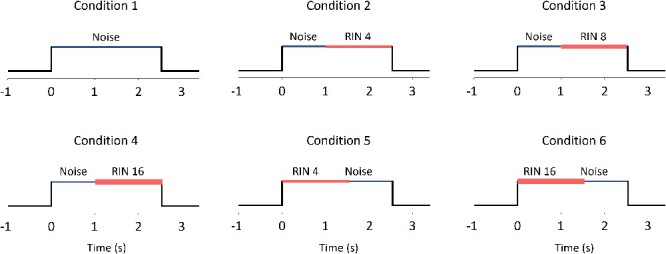
Diagrams of the stimuli used in the RIN iterations paradigm. Each subplot indicates a separate condition. RIN values show the number of delay-and-add cycles used to generate the stimuli, which results in a greater pitch percept as this number increases. Line thickness changes according to RIN value. Each trial consisted of 1 s of noise and 1.5 s of RIN.

Stimuli were presented binaurally via Etymotic ER4B earphones coupled with custom-made earmolds. Sounds were set to a comfortable listening level for each subject and were adjusted to be in the range of 45–55 dB above the audiometric threshold, and RMS levels were fixed for all stimuli within an individual participant. Subjects were awake and were instructed to passively listen to the sounds. Three different stimulus paradigms were implemented here (RIN iterations, RIN delays, and Harmonic Complex), all with 6 different conditions. MUA clusters were often recorded in 3 different paradigms on separate days or in different subjects due to time constraints; therefore, an across-subject design was used to conduct the analyses on the MUA recorded from the same contacts. In the RIN iterations paradigm, the first condition was a control, wherein each trial consisted of broadband noise with no iterations added. The other 5 of the stimulus conditions consisted of 1 s of a broadband noise period, which was either preceded or followed by RIN stimuli lasting 1.5 s (see Griffiths et al. 2010 for a more detailed description). We randomized the order of RIN associated stimuli to control for temporal-order effects, but we did not repeat the RIN 8 condition in the reversed condition in order to minimize time for our patients. RIN stimuli were generated using a delay-and-add algorithm with an increasing number of cycles (iterations), which results in an increasingly greater salience of the pitch percept (Yost et al. 1996). For this paradigm, the periodicity (i.e. pitch value) was set at either 128 or 256 Hz and was fixed for each recording. Figure 1 shows diagrams of these 6 conditions for this paradigm. In the RIN delays paradigm, stimuli consisted of 1 s of broadband noise followed by RIN with differing periodicity (8, 16, 32, 64, 128, and 256 Hz; 1.5 s duration). These stimuli are useful for determining the periodicity eliciting the maximum response of MUA, which is defined as the best rate. RIN stimuli in both paradigms were high-pass filtered above 800 Hz (using a 0-phase physically unrealizable filter in the frequency domain) and were normalized to the peak of the power spectral density, with broadband noise added below the 800 Hz cutoff. Finally, the third paradigm—Harmonic Complex—consisted of 1 s of Gaussian noise, which was either preceded or followed by a 1-s harmonic complex. The noise had the same passband as the harmonic complex. The fundamental frequency, *f*_0_ and harmonics of the harmonic complexes were *f*_0_ = 200 Hz (first to ninth harmonics: 200–1,800 Hz; or 10th–18th harmonics: 2,000–3,600 Hz), or *f*_0_ = 500 Hz (fourth to seventh harmonics: 2,000–3,500 Hz), with all components added in the sine phase. All conditions were combined with broadband noise (20 dB lower than harmonic complex tones) to mask distortion products. Diagrams for the RIN delays paradigm and harmonic complex are shown in Supplementary Figs. 1 and 2, respectively. Stimuli for all paradigms were ramped (5 ms on/off) and were presented with 50 repetitions per condition in a randomized interleaved order. RMS levels for noise and pitch-inducing stimuli were matched within each paradigm. Trials were separated by a 2-s period of silence.

#### Spike sorting

Prior to spike sorting, data were downsampled offline to 12 kHz and were denoised using the demodulated band transform (Kovach and Gander 2016). In order to threshold multiunit data from noise, spike sorting was performed for each recording block using the MountainSort algorithm (Chung et al. 2017), which offers a fast and fully automated approach to spike sorting by utilizing both principal component analysis and the ISO-SPLIT clustering algorithm (Magland and Barnett 2015). Briefly, data were first band-pass filtered between 300 and 6,000 Hz, whitened to decorrelate the data across channels, and then run through the MountainSort algorithm. Sorted spikes were imported into MATLAB (R2018b, MathWorks, Natick, MA, United States) for visualization and manual curation. For example, separate clusters of MUA were combined on each electrode (as extracted spike waveforms did not conform to a classical depiction of well-isolated single units with low variability), and any spuriously high amplitudes (>500 μV) were removed before epoching. All spike sorted data were then epoched around the stimulus for each condition, beginning 1 s before the first stimulus onset to 4 s after.

#### Analyses

Peri-stimulus time histograms (PSTHs) were produced for each auditory stimulus condition. Auditory responsive channels were defined as increasing their firing rate to the auditory stimulus by >3 SD above the mean baseline firing rate (−1,000 to −100 prestimulus) for a minimum of 25 ms at the onset of the stimulus. Responsiveness to pitch-like stimuli was determined using the same criteria against prestimulus baseline. Latencies were extracted from averaged PSTHs as the timing of the maximum response. For the RIN iterations paradigm, MUA clusters were classified as pitch-responsive if they showed a selective increase in firing to the RIN stimulus (>3.d. above prestimulus mean)—this was also examined in conditions where the transition was reversed (i.e. RIN to noise) in order to account for any time order or pop-out effects of the RIN stimulus and was used as a criteria for pitch-responsiveness. The relationship of firing rate with increasing iterations was also explored in this paradigm, as it was expected that with increasing iterations (i.e. salience), we would observe an increase in firing rate for MUA clusters that were pitch selective. Pitch selectivity for the RIN delays paradigm was defined as selective responsiveness (>3 SD above baseline) that was evident above the lower limit of pitch (~30 Hz; Krumbholz et al. 2000), while best rates were determined to the same stimuli as the periodicity of the RIN that evoked the greatest increase above baseline in firing within the first 500 ms of the RIN stimulus onset, the timing of which would encompass both the onset and the sustained response to the stimulus. The rate of RIN corresponds to the expected pitch frequency to be perceived (i.e. 256 RIN would correspond to a pitch frequency of 256 Hz). Therefore, rates of 8 and 16 Hz would not be expected to produce a pitch percept, as this is below the lower limit of pitch. For the harmonic complex paradigm, pitch MUA was defined as MUA clusters that responded preferentially (>3 SD above baseline) to a particular harmonic complex stimulus. We expected that the majority of MUA clusters would show maximal responses to the lower harmonics of the 200-Hz stimulus due to these being resolved by the cochlea and thus producing a stronger pitch percept, although we did not rule out that some MUA clusters may be responsive to higher harmonics, as these stimuli do still result in a pitch percept, albeit weaker.

Responses in all cases were confirmed by visual inspection of PSTHs. Channel locations were plotted on an MNI template of HG, with each of their MNI coordinates used to indicate their location along the axis of HG and with different symbols used to indicate whether MUA clusters showed selective responses to pitch-like stimuli or no selectivity at that location. We avoided any spatial binning or clustering of responses to maintain the benefit of spatial precision afforded by high impedance recordings. In order to display multiple MUA clusters that spatially overlapped, such as those that occurred across different sessions or paradigms, MNI *X* coordinates were shifted by 1 mm for the purposes of plotting on the template brain. However, the original MNI coordinates are displayed in Supplementary Table 1. Differences in proportions of pitch/nonpitch MUA clusters for the 3 different paradigms were tested for significance using the Freeman–Halton extension of the Fisher exact probability test (Freeman and Halton 1951). A repeated-measures 1-way ANOVA with Tukey post hoc test was performed to examine the change in firing rate across MUA clusters with increasing iterations of the RIN iterations stimuli when preceded by noise. For the purposes of consistency, conditions where noise followed the pitch-eliciting stimulus were not included in this test, as the onset response without a prior stimulus may have affected interpretation—that is, a neuronal response without an ongoing stimulus may be greater than that with an ongoing stimulus prior (for example, due to stimulus-related adaptation). To examine whether the likelihood of pitch responsiveness could be predicted by the distance of a contact from a putative pitch center, for each unique contact, we first calculated the Euclidean distance from the MNI coordinates provided in Patterson et al. (2002) (left MNI: [−55.3, −12.9, 1.5], right MNI: [57.2, −8.8, −1.9]) and then fitted a logistic regression model to these using custom-written MATLAB code, with pitch-responsiveness as the categorical outcome variable. To account for the potential presence of a pitch center anywhere along the axis, we extended this further by performing an additional analysis that involved fitting a quadratic model to the *X* and *Y* coordinates of all unique contacts (with the absolute value of *X* taken in order to merge across hemispheres) and performing a likelihood ratio test against an intercept-only model. Data are available from the corresponding authors upon reasonable request.

## Results

Figure 1 shows representations of the 6 conditions for the RIN iterations paradigm, wherein pitch salience is increased by increasing the number of delay-and-add cycles with a fixed delay period. We also recorded responses to 2 other paradigms: RIN delays, in which the periodicity of the RIN is varied by changing the delay period to assess the preferred rate of MUA clusters; harmonic complex, where the stimuli produce a percept of either a 200 or 500 Hz pitch (see Materials and methods for further details). For all 3 paradigms, pitch responsiveness was identified as a selective increase wherein firing rates increased by 3 SDs above the baseline mean in response to the pitch-evoking aspects of the stimuli (either RIN or harmonic complex).

### RIN iterations

**Fig. 2:**
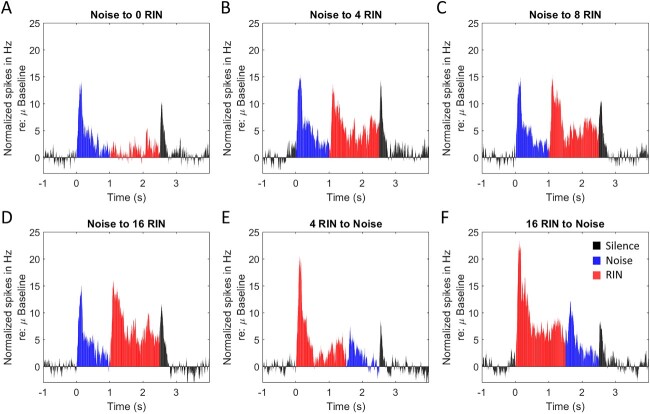
Grand mean PSTHs across all pitch-responsive MUA clusters (1-ms bins smoothed with a 25-ms window size moving average for display purposes), recorded in response to the RIN iterations paradigms (*n* = 26), with varying iterations (A-F). Grand mean values shown are normalized (via subtraction prior to smoothing) relative to the mean firing during the prestimulus baseline and each subplot represents a different condition. Data are averaged across 128- and 256-Hz stimuli. Red bars, RIN period (1 to 2.5 s A-D; 0 to 1.5 s E-F); blue bars, noise period (0 to 1 s A-D; 1.5 to 2.5 s E-F); black bars, silence period (<0 s and >2.5 s). Note that, for visualization, the *Y*-axis for this figure has lower bounds than Figs. 3 and 4, as this figure represents the grand mean across all clusters for this paradigm, while those are single examples and thus have less variability in response types.

Before examining the spatial distribution of pitch responses, we first examined the response patterns of MUA clusters by averaging across spatial locations along HG. Each panel in Fig. 2 represents the grand mean normalized PSTH of all pitch-responsive clusters of MUA (as defined above) to each condition of the RIN iterations paradigm (*n* = 26 MUA clusters, 50 trials per condition per MUA cluster), wherein the RIN stimulus had a fixed periodicity of either 128 or 256 Hz and only the number of iterations was incremented. This paradigm alters the salience of the pitch percept, and MUA clusters showed increased firing with increasing iterations, which is consistent with representing increasing pitch salience. Given the consistency of response patterns to the RIN iterations paradigm, after determining whether each MUA cluster was pitch responsive or not (>3 SD increase in firing rate above prestimulus baseline), it was feasible to average across all MUA clusters determined to be pitch responsive, for the purposes of depicting the data, unlike for the other 2 paradigms (wherein MUA clusters would be expected to show preferential responses to different conditions of the paradigm).

On average, there was an initial onset response to the onset of the initial noise stimulus (time 0 in the first 4 panels, peak response between 138 and 151 ms poststimulus) and an offset response to all the stimuli. When no iterations were added to the noise, there was generally only an onset to the initial noise (at 0 s) and then a sustained response to the ongoing noise (blue and red bars in Fig. 2A), which was followed by a fast offset response (peak at 58 ms poststimulus offset; final black bars in Fig. 2A). The peak response to the onset of the noise was between 128 and 156 ms when the noise was preceded by a pitch-eliciting stimulus (Fig. 2E and F). In 5 of the 6 conditions, noise was iterated with a delay-and-add algorithm to create the pitch-like percept (RIN). The peak onset responses to these RIN stimuli were between 111 and 114 ms when preceded by noise and did not systematically differ according to the salience of the pitch percept (Fig. 2B–D). When preceded by silence, the peak of the onset response occurred between 108 and 122 ms (Fig. 2E and F). In all these RIN conditions, a fast offset response was also present when the stimulus was followed by silence, with the peak occurring between 49 and 58 ms following the cessation of any auditory stimulation. The onset responses to the RIN stimuli were larger than the onset responses to the noise when both types of stimuli were preceded by silence despite being the same RMS sound level, with a peak Δ firing rate of 15.35 Hz for noise, 20.86 Hz for 4-RIN, and 23.39 Hz for 16-RIN. For the 16-RIN condition preceded by silence (i.e. the most responsive condition), mean baseline firing rates for grand mean MUA were 20.46 Hz (±1.15 SD). Mean firing rates within 250 ms of stimulus onsets for this condition were 34.85 Hz (±6.62 SD) for RIN and 28.65 Hz (±2.33 SD) for noise.

### RIN delays

**Fig. 3:**
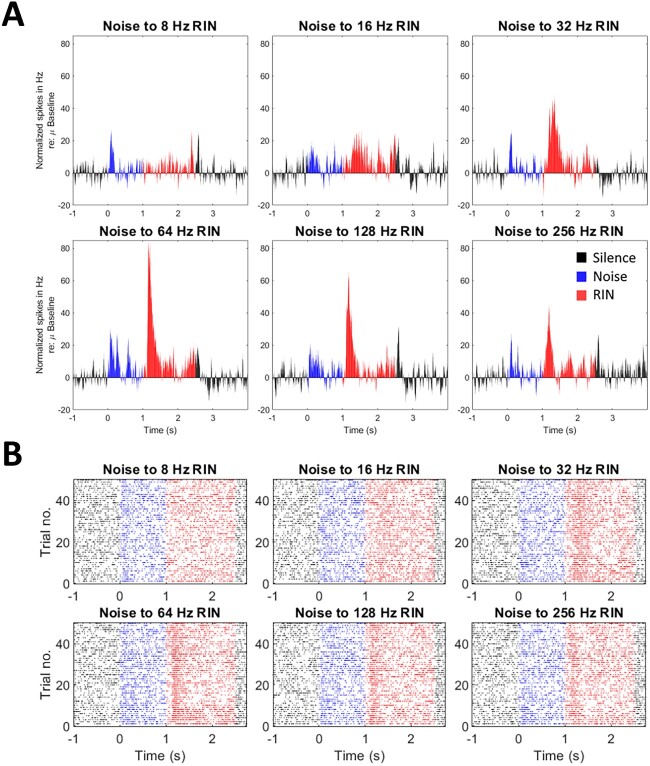
An example of normalized MUA PSTHs to the A) RIN delays paradigm, with corresponding B) spike time raster plots. Red colors represent RIN period (1 to 2.5 s), blue colors show noise period (0 to 1 s), and black colors show silence period (<0 s and >2.5 s). Each of the six panels for A and B represent a different condition, with each condition being a variation of the periodicity of the stimulus. This MUA cluster shows a best rate (i.e. greatest firing) of 64 Hz.

Figure 3A shows example MUA PSTHs to the 6 different conditions of the RIN delays paradigm (50 trials per condition), and the corresponding trial rasters are shown in Fig. 3B. This paradigm relates to the frequency of the subsequent percept. In this particular example, the 64-Hz stimulus elicited the greatest increase in firing for this MUA cluster (red bars in fourth panel), with a peak change of 84.29 Hz relative to baseline; the normalized response to the onset of noise for this same condition was 30.69 Hz. Thus, this MUA cluster would be determined to be pitch responsive with a best rate of 64 Hz.

A total of 29 MUA clusters (out of 42) were determined to be pitch responsive for these stimuli. Different MUA clusters showed different best rates. The number of MUA clusters with best rates for each of the frequencies above the lower limit of pitch were 3 for 32 Hz, 7 for 64 Hz, 3 for 128 Hz, and 16 for 256 Hz. These values are shown later as proportions in Fig. 5C, while individual tuning curves for each cluster (normalized to the maximal change in firing rate) are shown in Fig. 5D as well as with mean ± standard error overlaid in Supplementary Fig. 3. There was only 1 unit that showed a preferred rate of 16 Hz, but this was defined as nonpitch due to being below the lower perceptual limit of pitch. Nonetheless, in the current study, an overwhelming lack of units showing responsiveness to the 8 or 16 Hz stimulus argues against the idea that we are only looking at a continuum of responses to amplitude modulation here and are indeed examining responses that could underlie the pitch perception.

### Harmonic complex

**Fig. 4:**
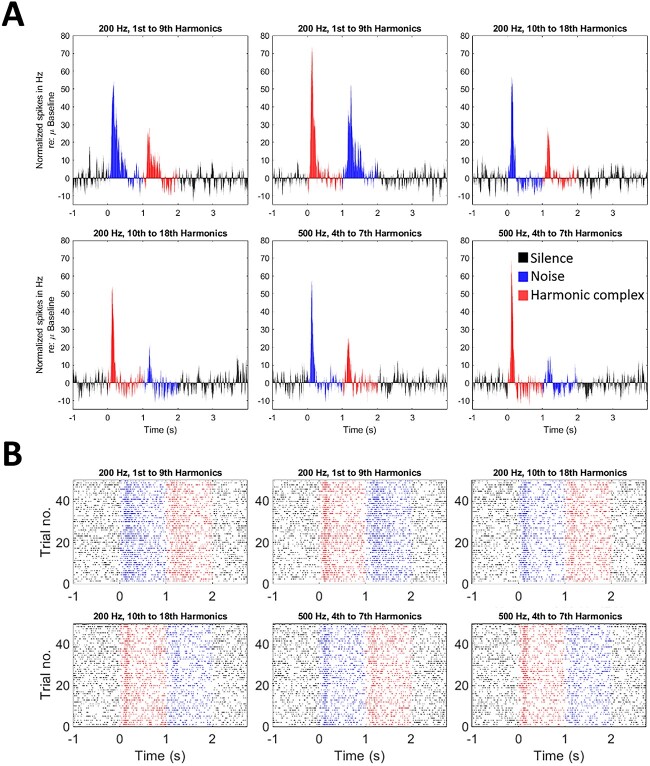
An example of normalized MUA PSTHs to the A) Harmonic Complex paradigm, with corresponding B) spike time raster plots. Red colors represent harmonic complex period (1 to 2 s odd panels; 0 to 1 s even panels), blue colors show Gaussian noise period (0 to 1 s odd panels; 1 to 2 s even panels), and black colors show silence period (<0 s and >2 s). Each of the 6 panels for A and B represent a different condition. From left to right, the first 2 panels in each subplot show responses to resolved harmonics for 200-Hz stimulus, the next 2 show higher harmonics for 200 Hz, and the last 2 are responses to the 500-Hz stimulus. This MUA cluster is an example of pitch-responsive MUA, showing a preferential response to the lower (i.e. resolved) harmonics of the 200-Hz stimulus.

Example PSTHs for a responsive MUA cluster to the harmonic complex stimuli (50 trials per condition) are shown in Fig. 4A, with corresponding raster plots in Fig. 4B. This example shows the strongest response to the resolved harmonics of the 200-Hz stimulus. The peak change relative to baseline for this condition was 74.84 Hz when preceded by silence (with a latency of 132 ms poststimulus onset; second panel in Fig. 4A) compared to a peak change of 59.04 Hz for Gaussian noise when preceded by silence (latency of 121 ms; fifth panel in Fig. 4). Interestingly, for the example presented in Fig. 4, this MUA cluster shows a clear onset response, but, then, often stimulus-specific suppression after this initial onset. This may indicate mixed response types for this MUA cluster, with ≥1 unit(s) being suppressed by the stimuli and other(s) showing mainly onset responses.

**Fig. 5:**
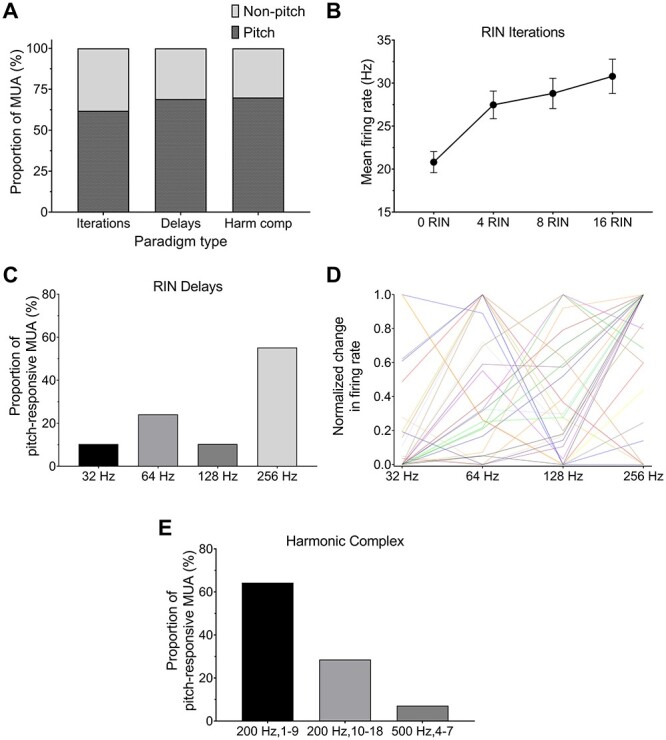
A) Proportion of auditory-responsive MUA recorded for each paradigm. Dark checked areas represent pitch-selective responses, and light gray areas show proportion of nonselective MUA. Harm comp, harmonic complex. B) Mean firing rate (Hz ± standard error) across MUA in response to increasing RIN values (*X*-axis) in the RIN iterations paradigm, averaged across the first 500 ms of stimulus presentation with noise preceding for each MUA. Higher RIN values relate to greater pitch salience. C) Proportion of best rates of pitch-responsive MUA in response to the RIN delays paradigm. D) Individual responses to RIN delays paradigm, normalized to the maximal change in firing rate for each pitch-responsive MUA cluster. E) Proportion of pitch-responsive MUA showing a maximal response to each of the harmonic complex stimuli.

A total of 14/20 MUA clusters showed pitch-selectivity using this paradigm. Proportions of preferred responses (i.e. maximal firing following baseline correction) in response to the harmonic complex paradigm are shown in Fig. 5E. The majority of MUA clusters (64%) showed selectivity for the lower, resolved harmonics of the 200 Hz stimulus, while 29% exhibited a maximal firing response to the higher harmonics and 7% responded with a maximal response to the 500-Hz stimulus. It is worth noting that the small proportion of MUA clusters showing a preferential response to the 500-Hz stimulus could be accounted for—at least in part—by fewer harmonics being included in this paradigm and thus resulting in a less salient pitch percept.

The total percentage of pitch and nonpitch MUA clusters across all subjects for the iterations, delays, and harmonic complex paradigms resulted in 26/42 (61.90%), 29/42 (69.05%), and 14/20 (70.00%) of MUA clusters showing pitch responses, respectively (Fig. 5A). We compared these proportions statistically to ensure that there was not 1 particular paradigm that was resulting in a higher or lower proportion of pitch-responsive MUA. As expected from the values above, there was no significant difference between the proportion of units classified as pitch responsive for 3 different paradigms (*P* = 0.74, Fisher’s exact probability test with Freeman–Halton extension). For the RIN iterations stimuli, across MUA, there was a clear increase in the firing rate corresponding to an increase in the salience of the stimulus (Fig. 5B). A repeated-measures 1-way ANOVA confirmed that there was a significant difference between the different iteration values (*F*(3, 75) = 39.23, *P* < 0.0001). As expected, given that these were pitch-responsive units, Tukey’s post hoc test highlighted that all RIN values >0 were significantly higher than 0 iterations (i.e. no pitch; adjusted *P* < 0.0001 for 4 RIN, 8 RIN, and 16 RIN compared to 0 RIN), while 16 RIN was also significantly higher than 4 RIN (adjusted *P* = 0.0058).

### Spatial distribution of pitch-responsive MUA

**Fig. 6:**
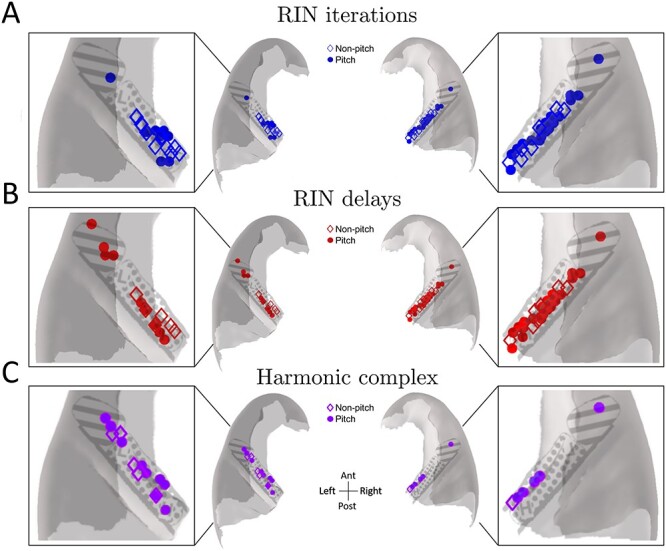
Distribution of response types for the 3 different paradigms: A) iterations (i.e. salience), B) delays (i.e. frequency), and C) harmonic complex. MUA responses are plotted on a top-down, axial template section of HG according to the MNI coordinates of their corresponding electrode contacts. Open diamonds represent nonpitch responses, and filled circles indicate pitch responses. HG has been approximately subparcellated into posteromedial HG (dotted) and anterolateral HG (lines) based on Wallace et al. (2002). Inset panels show magnified representations of response types overlaid on superior temporal plane.

To examine the spatial distribution of pitch and nonpitch responses, MNI coordinates of corresponding electrode contact locations were plotted on a template axial section of HG for both pitch and nonpitch selective MUA (Fig. 6). This figure shows that, regardless of the stimulus paradigm, there was no clear specific location with a greater abundance of pitch-responsive MUA, as pitch-responsive units were distributed throughout HG. Our coverage of the putative pitch region—which has been suggested to be located in anterolateral HG, mostly encompassing a region in nonprimary auditory cortex and slightly extending more medially into primary auditory cortex (Bendor and Wang 2006; Norman-Haignere et al. 2013)—was relatively limited, so we cannot rule out the possibility of a greater number/proportion of pitch-responsive units within this region. However, overall, these data are inconsistent with the concept of a single region that is unique in representing responses related to pitch in humans. Furthermore, we ran an exploratory analysis where we examined the likelihood of MUA being pitch responsive based on distance from a putative pitch center, which was calculated according to Euclidean distance from the coordinates provided in Patterson et al. (2002). If one were to assume that there was a particular region with a greater pitch representation, it is reasonable to suggest that the likelihood of observing pitch-responsive MUA would be predictable from a distance-based measure. Employing logistic regression, with the Euclidean distance of each contact from the putative pitch center of the corresponding hemisphere as the predictor variable and pitch-responsiveness as a categorical response variable, we found no indication that there was a greater likelihood of pitch responsiveness closer to the previously identified pitch region (β = 0.05, *P* = 0.30). Additionally, we examined whether a pitch center could be clustered or spatially located anywhere along the axis of HG by first fitting a quadratic model to the *X* and *Y* coordinates of all unique contacts and then performing a likelihood ratio test against an intercept-only model (see Materials and methods). The result of this analysis was also nonsignificant (*P* = 0.19), thus highlighting that we could not reject the null hypothesis of no spatial separation in whether an MUA cluster was pitch responsive or not.

**Fig. 7:**
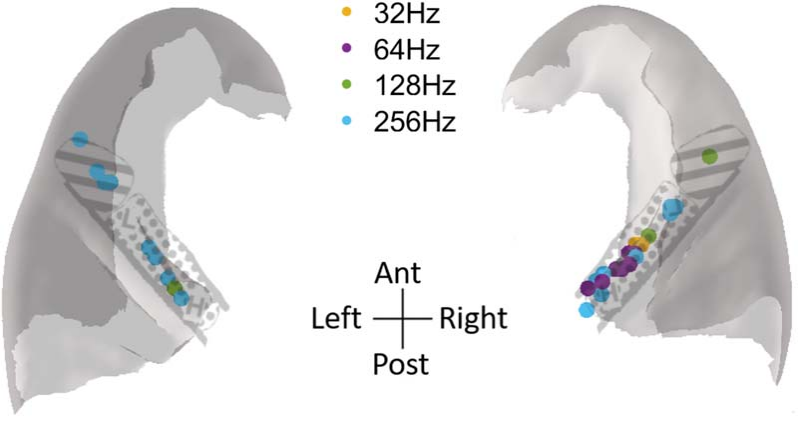
Distribution of best rates (in terms of periodicity, i.e. pitch tuning) in response to the delays paradigm, plotted on a top-down, axial template section of HG. Subparcellations of posteromedial (dotted) and anterolateral HG (lines) from Wallace et al. (2002).

We also sought to determine whether there was any clear spatial distribution to the preferred periodicities of the MUA, which we termed as “best rate” (Fig. 7). Consistent with findings from macaques (Kikuchi et al. 2019), there was no clear map of best rates, as these were distributed throughout HG. However, it should be noted that there may be some degree of sampling bias, as the majority of MUA (56.52%) responded maximally to the 256 Hz stimulus. Although there was a cluster of responses with a best rate of 64 Hz located in posteromedial HG on the right, it is possible that this also represents some degree of sampling bias, as this area still had other MUA with different best rates (e.g. 256 Hz). Indeed, this sampling bias for right HG is something that is further highlighted by Fig. 6B.

**Fig. 8:**
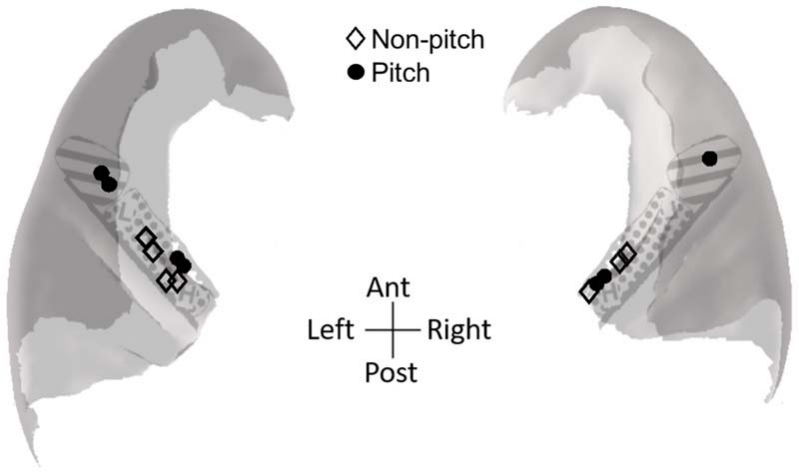
Distribution of pitch responses by using a conservative approach with a requirement that MUA had to show pitch-like responses to both harmonic complex stimuli and RIN paradigm(s). Subparcellations of posteromedial (dotted) and anterolateral HG (lines) from Wallace et al. (2002).

Finally, we performed an additional conservative analysis on a subset of data where we had responses to both harmonic complex and RIN stimuli at a single location, with a requirement that MUA had to show pitch-like responses both harmonic complex stimuli and RIN iterations/delays paradigms (or both, if recorded) to be considered as pitch responsive. For this subset of 14 contacts, 7 were defined as being pitch responsive under this new criterion (50%). The coordinates, along with the response types, for this analysis, are displayed in Fig. 8. Although this resulted in a very sparse sampling of HG due to the restricted number of responses we had to multiple paradigms, pitch responses were still not isolated to a putative pitch region, thereby arguing against the existence of a sole region in anterolateral HG responsible for the processing of pitch.

## Discussion

The current study is the first of its kind in humans, wherein we have benefitted from the spatial precision of high-impedance electrodes to identify the locations of multiunit responses to pitch-like stimuli as well as the temporal precision of electrophysiology to determine peak response latencies. These responses were distributed throughout HG, rather than confined to a particular region, thereby inconsistent with the idea of a single specialized region for pitch in humans.

### Distributed human neural responses to the pitch percept

In this study, we have defined MUA associated with pitch in 3 different ways: (i) responses to RIN that increase with pitch salience; (ii) responses to RIN that are in the range of rates associated with a pitch percept; and (iii) responses to harmonic complexes associated with a pitch percept. In all 3 paradigms, we see responses that are distributed within auditory cortex and not confined to a specific region. The type of stimulus used to generate a pitch percept likely has an effect on the interpretation of results from different studies. In the current study, we attempted to mitigate this somewhat by examining responses to 2 different but commonly used types of stimuli—RIN and harmonic complex stimuli. As mentioned previously, in MRI studies, the type of control stimulus greatly affects interpretation of results, as the analysis often requires subtraction by design (discussed in Barker et al. 2012 and Bendor 2012). Examining spiking activity allows for this requirement to be eased, as absolute changes in firing rate over time can be observed, while parametrically adjusting the strength of the pitch stimulus, even prior to comparing to another condition, meaning that we can look at more subtle changes in firing rates over time to determine responsiveness. We also were able to examine the pitch specificity more robustly by reversing the presentation order of the stimuli. Therefore, we can be reasonably confident that the responses seen here are not a consequence of either the type of control stimulus used or other properties of stimulus presentation (e.g. time order effects).

Moreover, the parametric design afforded by the 2 RIN paradigms increased our ability to examine pitch responsiveness in greater detail—that is, by varying either the salience or the frequency of the resultant percept. In an fMRI study examining the specificity of pitch responsiveness in a region of planum temporal, it was found that the magnitude of this response did not covary with pitch salience (Barker et al. 2011). It is plausible that such a finding may be due to the sensitivity of the recording technique—indeed, while we did observe increasing responsiveness in pitch MUA with increasing pitch strength, across MUA, these results were fairly subtle, with only ~11% change in peak firing rate between the least and most salient stimuli (Fig. 5B).

### Stimulus considerations

It is notoriously difficult to design experiments to define responses to pitch-associated stimuli and control stimuli with no associated pitch and a similar acoustic structure. This is a particular concern for the RIN iterations paradigm in which the delay-and-add algorithm produces slow spectrotemporal fluctuations that are associated with timbral changes as well as changing the pitch salience (Barker et al. 2012). In this experiment, we have also varied the pitch value in the delays paradigm and demonstrated responses that respect the lower limit of pitch. The spectrotemporal fluctuations in the stimulus, and the associated change in the timbral percept, do not respect the lower limit of pitch in this way. In the harmonic complex paradigm, we have compared these with spectrally matched noise, but the harmonic complexes contain spectral features (especially when low resolved harmonics are used) and temporal features (the regularity) that are not present in the control noise. In this study, we have defined responses to 3 different types of pitch-associated stimuli that are parsimoniously explained as pitch-evoking stimuli and responses. We find that the responses to all 3 manipulations are clearly distributed across auditory cortex, supporting the hypothesis of a distributed pitch-associated neuronal response in human auditory cortex.

### Comparison with animal models

Animal studies have investigated pitch responses in the auditory cortex of several species, sometimes with seemingly conflicting results. In ferrets, cortical responses indicative of pitch processing have been shown to be distributed across auditory fields (Bizley et al. 2009; Walker et al. 2011). Using high-field fMRI in cats, Butler et al. (2015) found that responses to RIN stimuli (compared to narrowband noise) were not present in the subdivisions relating to the core auditory cortex (A1 and anterior auditory field) but were instead unique to regions further upstream (posterior auditory field and A2). Bendor and Wang (2005) demonstrated regional selectivity located in anterolateral A1 and extending slightly further medially in marmosets, while Kikuchi et al. (2019) found responses distributed throughout the macaque auditory cortex. Species differences may play a role in some disparities. Indeed, it has recently been demonstrated that there are fundamental differences in the neural representation of harmonic stimuli between humans and macaques (Norman-Haignere et al. 2019), suggesting some degree of unique specialization in humans. With respect to this, Walker et al. (2019) discussed across-species differences in pitch perception in the context of variations in cochlear filtering.

It is also reasonable to assume that the criteria used for the definition of pitch responsiveness may explain the discrepancies between the different studies. In Bendor and Wang (2005), for example, a key requirement was that neurons recorded from marmosets responded similarly to harmonic complex stimuli and pure tones of a similar frequency. Due to time constraints with patients, we often did not obtain the pure tone responses on the same day for MUA in the current study, so we felt that it was inappropriate to apply this criterion throughout. However, we have explored this in an additional analysis (Supplementary Fig. 4) and found that contacts with pitch-responsive MUA that also responded to pure tones of a similar frequency did not spatially localize to a single region. Likewise, this was also examined in Kikuchi et al. (2019) in macaques, and no spatial specificity was found even when applying this criterion. Moreover, as mentioned in this latter study, it is feasible that there could be separate mechanisms for the processing of harmonic complex stimuli and pure tones, so this requirement may be overly restrictive**.**

An important limitation of the current study is that our sampling is sparse compared to that observed in fMRI studies. This is a necessary limitation of the intracranial methodology, as sampling is based primarily on the clinical need of the patients and not all contacts provided identifiable MUA. Therefore, as highlighted in the Results section, one cannot rule out a greater representation of pitch responsiveness within a particular area of HG. However, by showing any pitch-responsive MUA outside of the putative pitch center—which we have done here—we have ruled out the possibility that pitch responses are localized solely to a singular pitch region and are accounted for the possibilities of differences in spatial precision between human and animal studies. Indeed, our MUA is likely precise to within ~50–300 μm (Henze et al. 2000; Buzsaki 2004). Furthermore, these results are consistent with Kikuchi et al. (2019), wherein a greater sampling of auditory cortex was obtained. Finally, although the spatial precision was lower than shown here, results from LFPs across all channels from these same patients and others also highlighted a lack of spatial specificity in pitch representation (Gander et al. 2019).

The proportion of pitch-responsive units here is higher than that found in macaques (~20%; Kikuchi et al. 2019), although similar to proportions of voxels found in some auditory cortex regions in an imaging study utilizing RIN (Hall and Plack 2009). In the Kikuchi et al.’s (2019) study, the criteria for pitch-responsiveness were somewhat stricter than that employed here for our full dataset, as multiple paradigms were collected in the same units. When we applied these same stricter criteria, while we had a considerably reduced dataset in terms of sample size (due to not recording multiple paradigms to all units), we did indeed see a lower proportion of contacts classified as pitch responsive. Nonetheless, as reported in the previous Kikuchi et al.’s study, when employing stricter criteria, pitch responses in macaques and in this reduced dataset were not restricted to a single region within the anterolateral auditory cortex, which is consistent with our main finding here.

## Conclusion

We have successfully demonstrated pitch responsiveness in the neuronal spiking activity along the axis of HG, with sensitivity to both salience and pitch frequency. These responses were distributed throughout all regions examined rather than being restricted to a particular area. While this aspect apparently conflicts with some fMRI and marmoset studies, it is consistent with other human studies utilizing MRI (Hall and Plack 2009; Allen et al. 2022) and LFP recordings (Griffiths et al. 2010; Kumar et al. 2011; Gander et al. 2019) as well as macaque (Kikuchi et al. 2019) and ferret studies (Walker et al. 2011). As discussed in Kumar and Schönwiesner (2012), the examination of MUA in humans is a useful step in bridging the gap between human and animal data. Future studies examining connectivity patterns between regions beyond and including HG would be beneficial in understanding the distributed nature of pitch processing and in exploring in greater detail the roles of various regions, such as focusing on the response types (e.g. sustained responses) to the different pitch-inducing stimuli.

## Supplementary Material

Supplementary_Figure_1_with_FRAs_bhad186

Supplementary_Figure_2_white_background_bhad186

Supplementary_Figure_3_white_background_bhad186

Supplementary_Figure_4_bhad186

Supplementary_Table_1_revision_bhad186

Supplementary_Data_NEW_bhad186
