# Supplementary figures and images for: Distribution of multiunit pitch responses recorded intracranially from human auditory cortex
Source: Cereb Cortex. 2023 May 26;33(14):9105–16. doi: 10.1093/cercor/bhad186 (PMC10350829; doi:10.1093/cercor/bhad186)

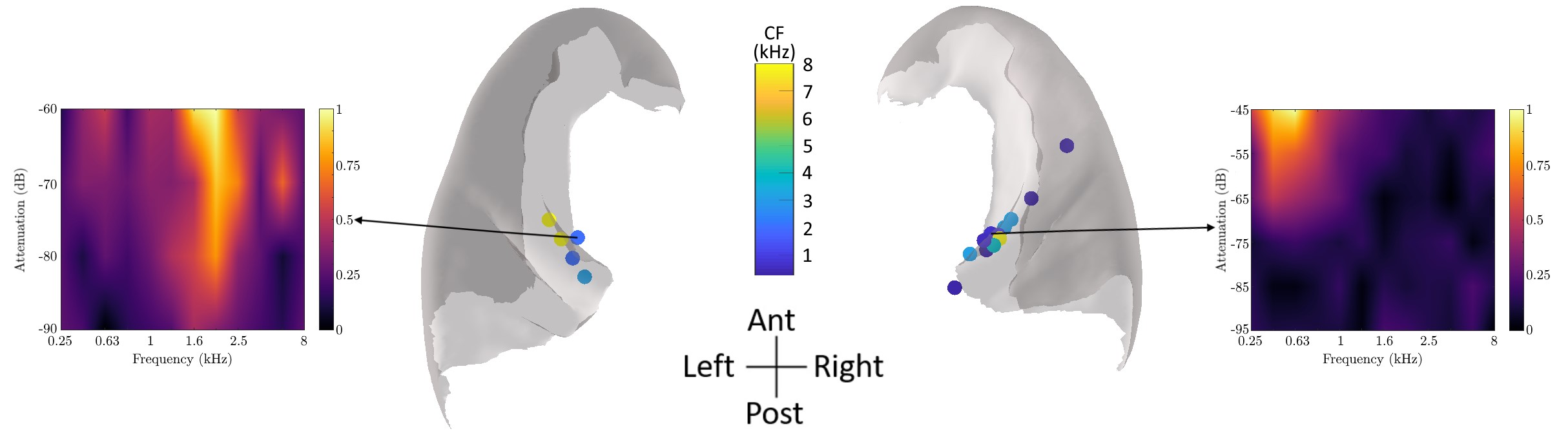

Supplement: Supplementary_Figure_1_with_FRAs_bhad186 [file supplementary_figure_1_with_fras_bhad186.jpeg]

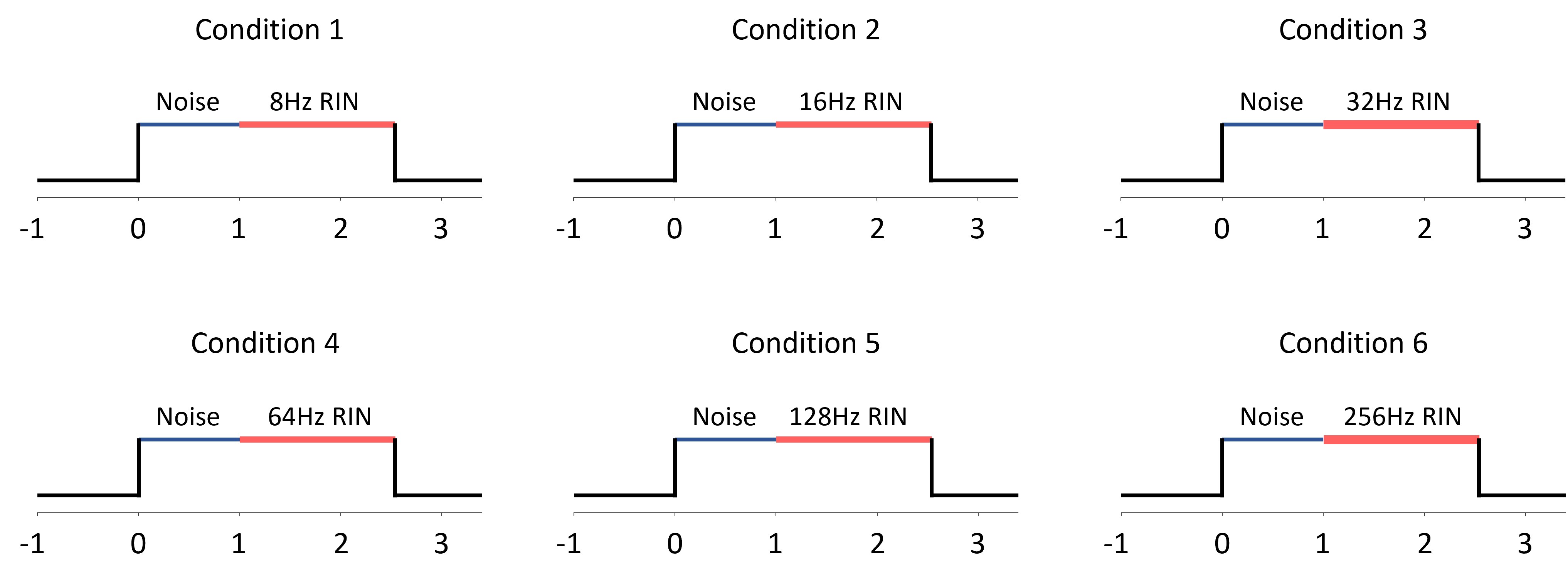

Supplement: Supplementary_Figure_2_white_background_bhad186 [file supplementary_figure_2_white_background_bhad186.jpeg]

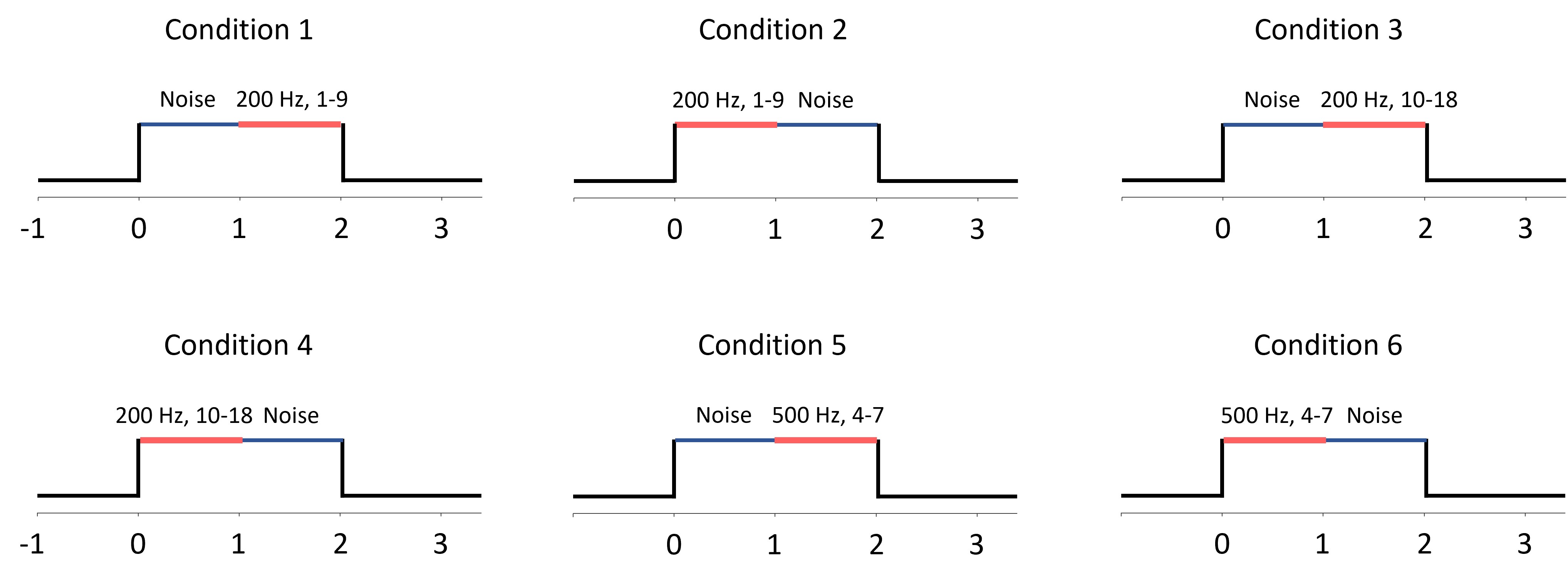

Supplement: Supplementary_Figure_3_white_background_bhad186 [file supplementary_figure_3_white_background_bhad186.jpeg]

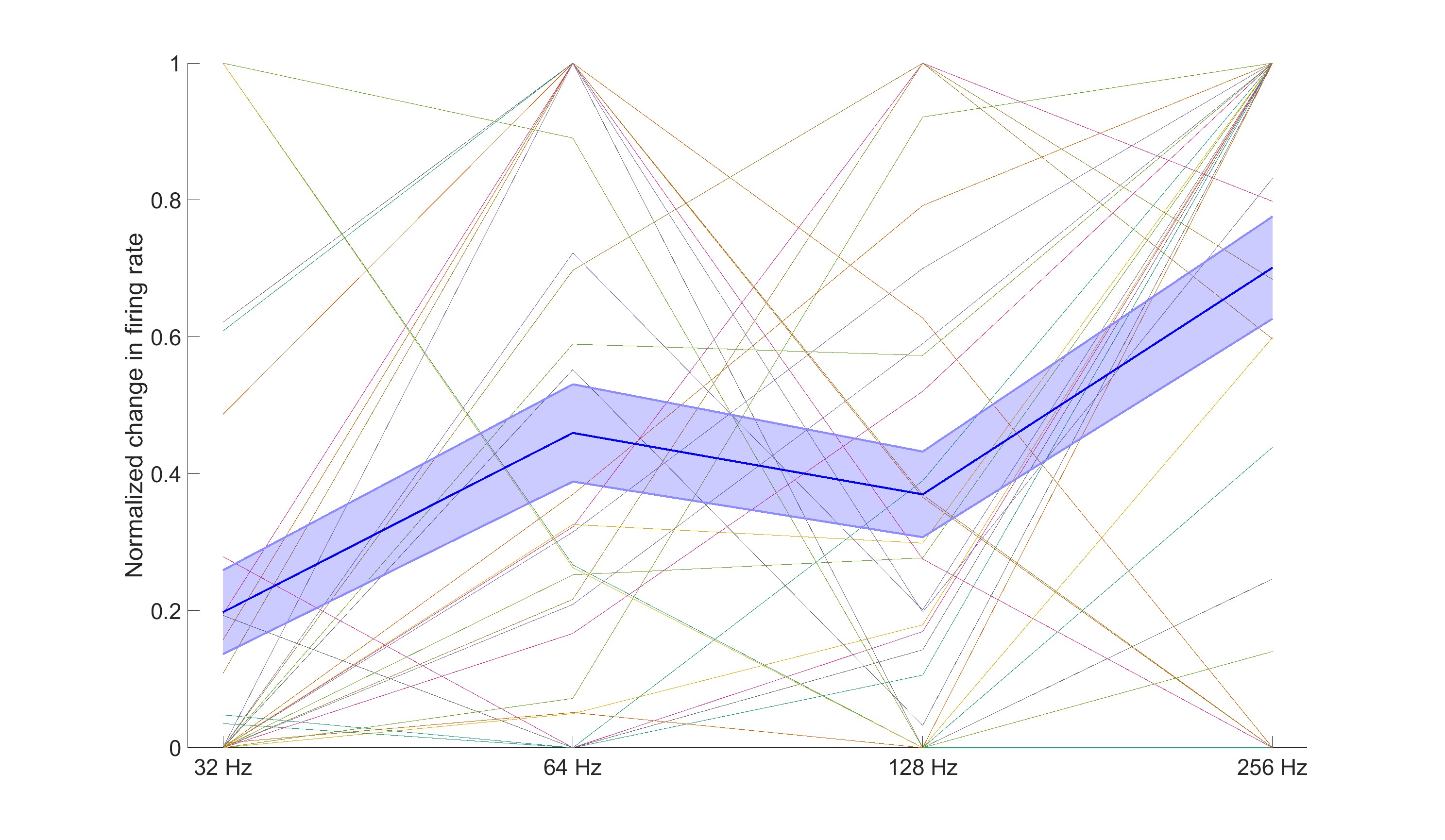

Supplement: Supplementary_Figure_4_bhad186 [file supplementary_figure_4_bhad186.jpeg]
