# Supplementary material for: Distribution of multiunit pitch responses recorded intracranially from human auditory cortex
Source: Cereb Cortex. 2023 May 26;33(14):9105–16. doi: 10.1093/cercor/bhad186 (PMC10350829; doi:10.1093/cercor/bhad186)
Supplement: Supplementary_Data_NEW_bhad186 [file supplementary_data_new_bhad186.docx]

**
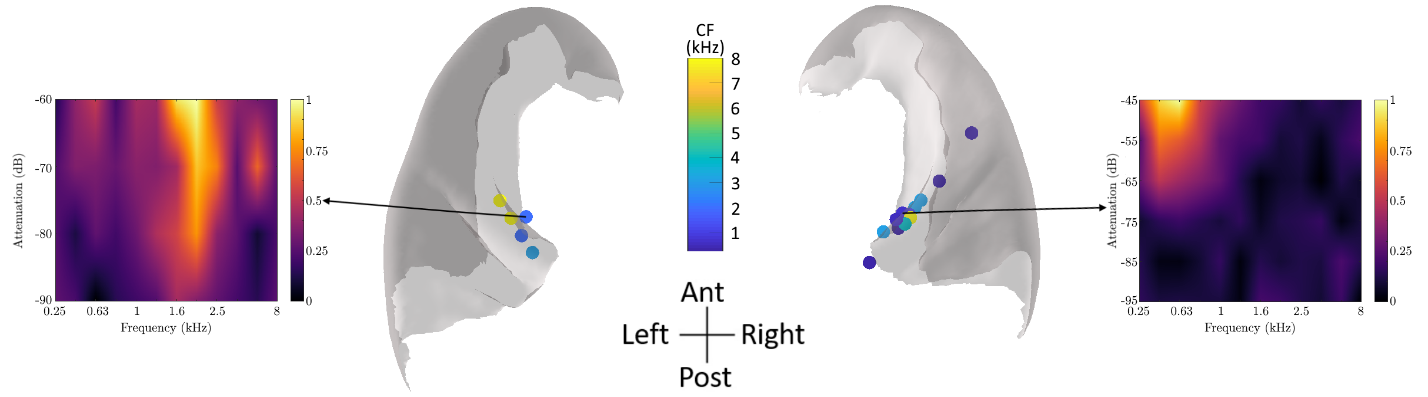
**

**Supplementary Figure 1. Center figure shows distribution of pitch-responsive contacts across all paradigms, wherein MUA also showed a significant response to pure tones of a similar frequency (within 1 octave). Contacts are color-coded according to their characteristic frequency (CF) determined by frequency response areas and PSTHs, constructed from responses to tones of varying frequency and amplitude (n = 17 unique contacts). Examples of these frequency response areas – normalized according to maximum firing rate – are shown either side. Both of these examples showed responses to pure tones of similar frequencies to the pitch-eliciting stimuli, though one had a higher CF (left) and had a lower CF (right). Note that a number of contacts had MUA recorded from them across different sessions in response to the pitch stimulus paradigms.**

**
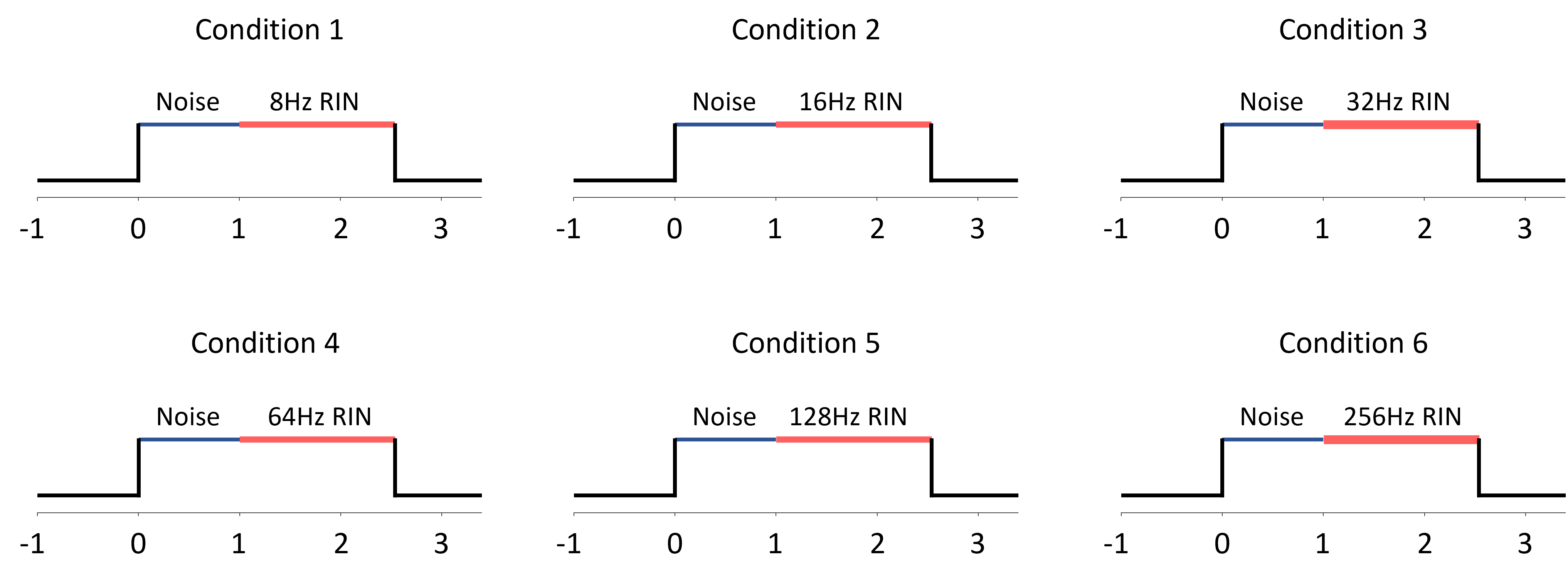
Supplementary Figure 2. Diagrams of the stimuli used in the RIN delays paradigm. Each subplot indicates a separate condition. RIN values show the periodicity of the stimuli. Each trial consisted of 1 second of noise and 1.5 seconds of RIN.**

**
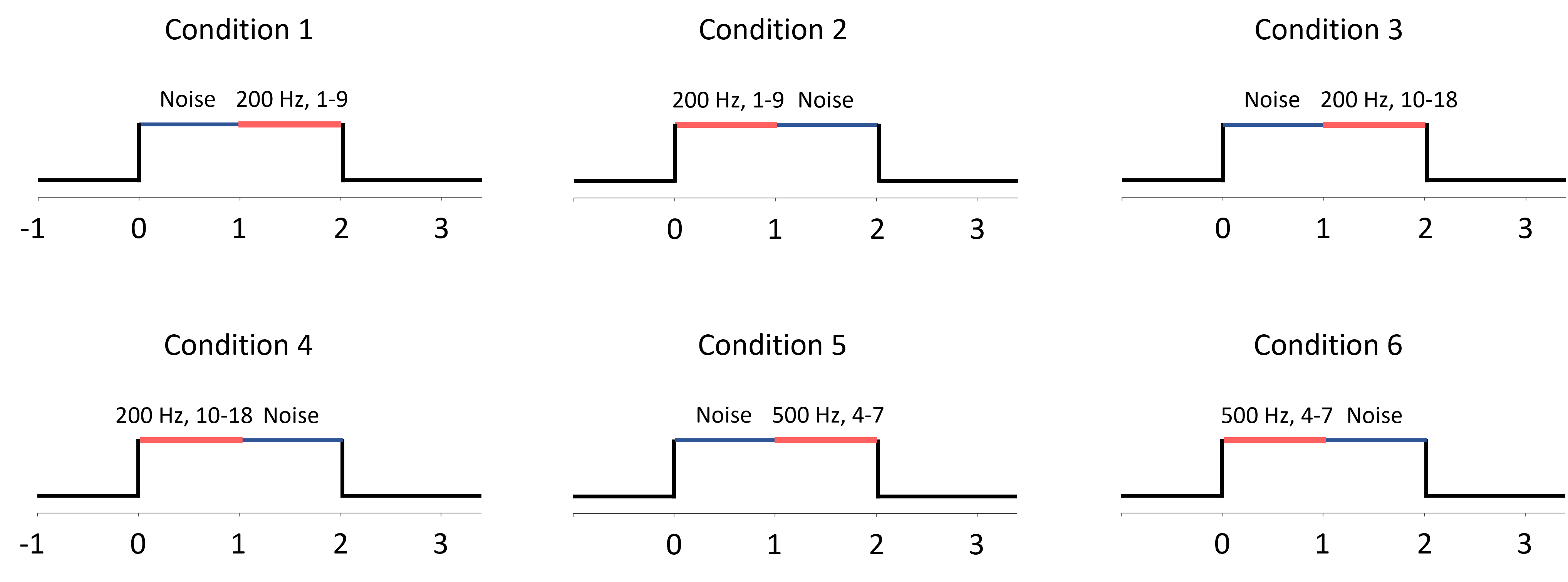
**

**Supplementary Figure 3. Diagrams of the stimuli used in the Harmonic Complex paradigm. Each subplot indicates a separate condition. Values in Hz relate to the missing fundamental of the stimulus, whilst following values indicate the harmonic components included (e.g. 1-9 indicates the first to 9^th^ harmonics). Each trial consisted of 1 second of noise and 1 second of harmonic complex stimuli.**

**
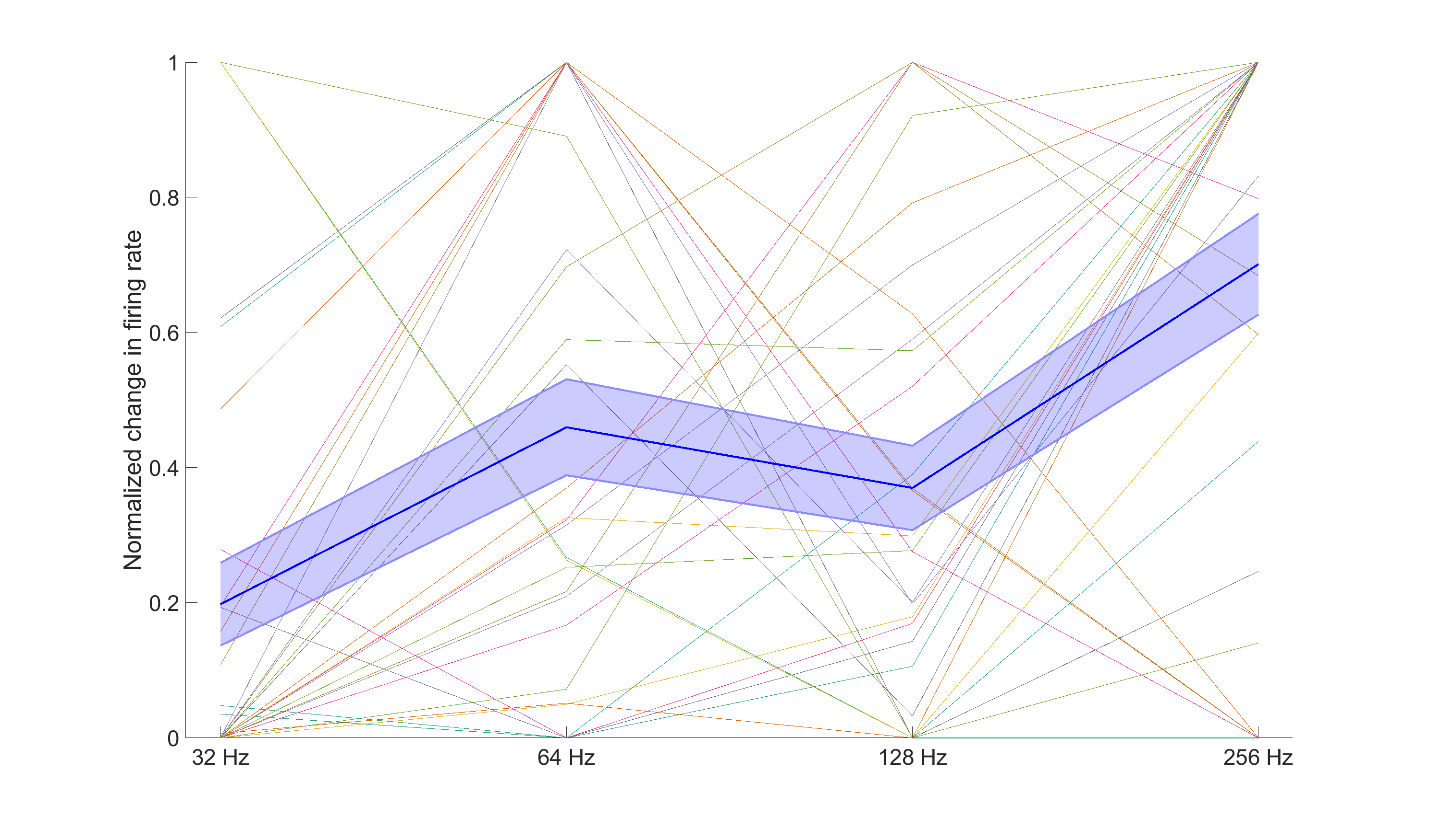
Supplementary Figure 4.** **Individual responses to RIN delays paradigm, normalized to the maximal change in firing rate for each pitch-responsive MUA cluster, along with mean (± standard error) across all clusters overlaid.**

**Supplementary Table 1**

Excel file containing block numbers, channel numbers, block type, response type and MNI coordinates.
